# Dual-path deep learning framework for accurate and interpretable brain tumor diagnosis

**DOI:** 10.1186/s12911-026-03367-7

**Published:** 2026-02-26

**Authors:** Reham F. Haroun, Hatem A. Khater, Mohamed A. Mohamed, Mohamed G. Abdelfattah

**Affiliations:** 1 Department of Electronics and Communications Engineering, Faculty of Engineering, Mansoura University, Mansoura, 35516 Egypt; 2 Faculty of Engineering, Horus University, New Damietta, Damietta, Egypt; 3 Faculty of Artificial Intelligence, Horus University, New Damietta, Damietta, Egypt

**Keywords:** Brain tumor diagnosis, Magnetic resonance imaging (MRI), Deep learning framework, Content-based image retrieval (CBIR), GhostNetV3, Deformable convolution

## Abstract

Existing frameworks for brain tumor diagnosis often focus on standalone classification or retrieval tasks, limiting clinical interpretability and failing to leverage complementary diagnostic insights. To address this, we propose a novel dual-path deep learning framework that synergistically integrates tumor classification with content-based image retrieval (CBIR). Our approach uniquely combines a lightweight GhostNetV3 backbone with deformable convolutions and a decoupled fully connected (DFC) attention mechanism to simultaneously optimize feature extraction for both tasks. This integration enables dynamic adaptation to irregular tumor morphologies while retrieving visually similar cases, bridging the gap between automated predictions and actionable clinical context. Evaluated on a public T1-weighted contrast-enhanced MRI dataset (233 patients, 3,064 images), the framework achieves state-of-the-art performance: 99.71% classification accuracy (precision/recall/F1 > 0.99) and 97.74% mean average retrieval precision (Prec@10: 99.78%). We further introduce the Classification-Retrieval Agreement Score (CRAS), a novel metric quantifying alignment between classifier predictions and retrieved cases, with a mean score > 0.96 demonstrating robust diagnostic consistency. By enhancing accuracy, interpretability, and computational efficiency, this work advances the clinical viability of AI-driven brain tumor diagnosis.

## Introduction

Brain tumors represent a significant health challenge, encompassing a diverse range of intracranial growths from benign to highly malignant [1]. Magnetic Resonance Imaging (MRI) plays an indispensable role in non-invasive diagnosis due to its exceptional anatomical resolution and sensitivity to subtle tissue changes, crucial for identifying common tumor types like gliomas, meningiomas, and pituitary adenomas [2]. While histopathological analysis remains the gold standard for definitive diagnosis, its invasive nature underscores the critical need for robust and reliable non-invasive alternatives. Manual MRI interpretation is inherently time-consuming, subjective, and prone to inter-observer variability, driving the demand for accurate and efficient automated diagnostic systems [3].

Traditional machine learning (ML) approaches in medical imaging often rely on handcrafted radiomic features (e.g., texture descriptors, intensity histograms), which inherently limit their ability to capture the nuanced spatial relationships and subtle morphological characteristics crucial for accurate tumor classification [4]. In contrast, deep learning (DL), particularly convolutional neural networks (CNNs), has profoundly influenced neuro-oncological image analysis by providing automated hierarchical feature extraction. Architectures such as ResNet [5], VGG16 [6], and hybrid models [7, 8] have demonstrated robust performance in tasks ranging from tumor segmentation [9] to subtype classification [6] and malignancy prediction [10]. Despite these advances, standalone classification models suffer from two critical limitations: (1) their black-box nature reduces clinician trust, and (2) they lack contextual support for differential diagnosis [11, 12].

Content-based image retrieval (CBIR) addresses these limitations by enhancing interpretability through retrieval of visually similar cases [13]. Early systems for brain tumors relied on handcrafted descriptors like bag-of-visual-words [14], tumor margin features [15], and spatial layout information [16]. While these methods showed initial promise, they struggled to capture clinically relevant anatomical variations due to their reliance on low-level features like Haralick textures and SIFT descriptors [17, 18]. Subsequent efforts like adaptive spatial pooling [19] reduced manual feature engineering but failed to fully exploit end-to-end deep learning. Modern systems leverage transfer learning [20, 21] and fine-tuning [22] to bridge the semantic gap between low-level features and high-level diagnostic understanding [23]. CNN-derived representations [24, 25] capture subtle morphological variations, improving diagnostic validation [14], rare subtype identification [19], and treatment planning [26]. Recent innovations include federated learning for privacy preservation [27], fusion of textural and visual features [28], and adaptive margin loss for robust similarity learning [29]. Novel techniques like 2D slice embeddings [30], hybrid meta-heuristic optimization [31], and attention-deep hashing [32] further demonstrate the field’s rapid evolution.

Recent review studies further emphasize that, despite significant progress in deep learning-based brain tumor analysis, challenges related to interpretability, clinical trust, and integration into decision-support workflows remain unresolved [33].

Despite these advancements, critical gaps persist in the recent literature (2023–2025). Most state-of-the-art approaches continue to focus on standalone brain tumor classification, achieving high predictive accuracy but offering limited interpretability and contextual support for clinical decision-making. For instance, recent studies by Disci et al. [34] and Babar et al. [35] demonstrate strong classification performance using transfer learning and attention-guided CNN architectures. Similar trends are reported in other recent works employing deep convolutional, ensemble, and hybrid deep learning models for MRI-based brain tumor classification across diverse imaging settings [36, 37]. However, these models predominantly function as black-box predictors and do not provide comparative visual evidence to support diagnostic decisions.

Conversely, a separate line of recent research has focused on content-based image retrieval (CBIR) as an independent diagnostic aid. While CBIR systems have shown promise in retrieving visually similar radiological cases using deep feature embeddings, they are typically designed independently of diagnostic classifiers and do not exploit the discriminative power of jointly learned representations. As highlighted by Denner et al. [38], modern CBIR systems in radiology emphasize feature generalization and retrieval accuracy yet lack explicit integration with tumor classification pipelines.

This separation between classification and retrieval leads to duplicated computation, suboptimal feature alignment, and an inability to quantify diagnostic consistency between predicted labels and retrieved reference cases. These limitations underscore the need for a unified framework that jointly optimizes tumor classification and CBIR within a single architecture, ensuring feature-level alignment, interpretability, and computational efficiency.

To bridge these gaps, we propose a dual-path deep learning framework that unifies tumor classification and CBIR through a shared, lightweight GhostNetV3 backbone [39] enhanced with deformable convolutions [40] and a decoupled fully connected (DFC) attention mechanism. Unlike sequential or task-isolated approaches [26], the proposed model jointly optimizes classification and retrieval, enabling mutual refinement of feature representations that are simultaneously discriminative for diagnosis and semantically meaningful for retrieval. Furthermore, we introduce the Classification–Retrieval Agreement Score (CRAS), a novel metric that quantitatively measures the internal consistency between predicted tumor labels and retrieved reference cases—an aspect largely overlooked in existing studies. This combination of joint learning, explicit alignment measurement, and parameter efficiency distinguishes the proposed framework from recent state-of-the-art methods and enhances its suitability for clinical decision support. Our key contributions are as follows:

- A dual-path architecture integrating tumor classification and CBIR via a shared GhostNetV3 backbone, enhanced with deformable convolutions and DFC attention for precise brain tumor characterization.
- Superior performance over existing recent schemes on the Cheng MRI dataset [41]: 99.71% classification accuracy (precision/recall/F1 > 0.99) and 97.74% mean average retrieval precision (Prec@10: 99.78%).
- The CRAS, a novel metric quantifying the alignment between classifier predictions and retrieved visual evidence, achieves a mean score exceeding 0.96. This addresses a critical gap in validating diagnostic consistency for clinical decision support systems.
- Computational efficiency achieved through a parameter-efficient shared backbone design and minimal preprocessing, facilitating potential real-world clinical deployment.

## Methodology

**Fig. 1:**
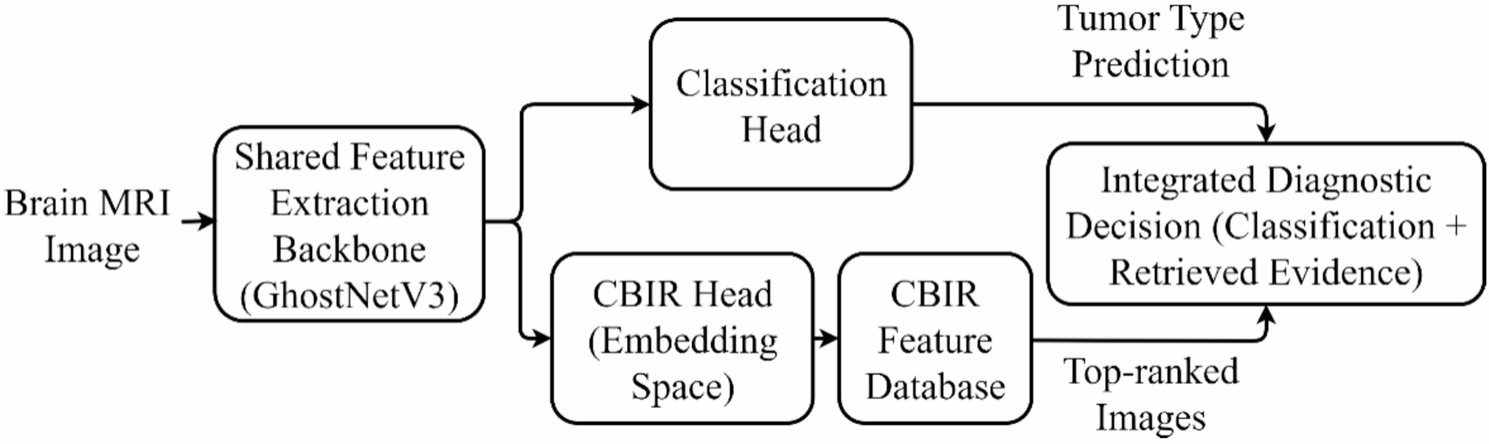
Overview of the proposed dual-path diagnostic framework. A shared GhostNetV3 backbone feeds parallel classification and CBIR branches, enabling automated tumor prediction alongside retrieval of visually similar reference cases to support case-based clinical decision-making

The proposed dual-path deep learning framework for brain tumor diagnosis is depicted in Fig. 1. This innovative architecture seamlessly integrates CBIR with tumor classification, creating a comprehensive and automated diagnostic system. The framework utilizes a shared feature extraction backbone to process input MRI images, which then branches into two parallel pathways: one for tumor classification and the other for retrieving visually similar images from a database using CBIR. This dual-path design facilitates both direct diagnosis and the provision of contextual visual information, enhancing clinical decision-making. The following subsections detail each component.

### Dataset and preprocessing

We utilize the publicly available Cheng dataset [41] for training and evaluation. This dataset comprises 3,064 T1-weighted contrast-enhanced MRI slices from 233 patients. The dataset categorizes these slices into three distinct brain tumor types: glioma, meningioma, and pituitary tumor. Table 1 details the distribution of patients and images across each class. Each MRI slice has dimensions of 512 × 512 pixels and a pixel resolution of 0.49 mm × 0.49 mm, consistent with standard clinical practice. The use of 2D slices, as opposed to 3D volumes, significantly reduces computational demands, enabling more efficient training and inference.

**Table 1:** Dataset summary

Tumor Type	Number of Patients	Number of Slices
Glioma	91	1,426
Meningioma	82	708
Pituitary	60	930
Total	233	3,064

All images undergo preprocessing prior to model training and evaluation. We resize images for compatibility with the CNN input layer, followed by intensity normalization using Min-Max scaling to the range [0,255]. This enhances image contrast and improves the visibility of subtle but clinically significant features, such as tumor boundaries and textures. Finally, we convert pixel data to an unsigned 8-bit integer (uint8) format for efficient storage.

To further enhance model generalization and mitigate overfitting, we apply several data augmentation techniques during training. These include random cropping, horizontal and vertical flipping (each with a probability of 0.5), and random brightness and contrast adjustments (0.2 probability). Additional augmentations include random rotation (± 50 degrees, 0.5 probability), Gaussian blur (0.2 probability), and affine transformations. The affine transformations combine random shifts (0.0625 of image dimensions), scaling (up to 10%), and rotation (± 45 degrees). These augmentations artificially expand the training dataset’s diversity, improving the model’s robustness to variations inherent in clinical MRI data.

All data augmentations were applied after patient-wise data partitioning and exclusively to the training split within each cross-validation fold. This protocol ensures that no augmented slices from a given patient appear in the validation or test sets, preventing information leakage despite the inter-slice correlations inherent in MRI volumes. As the proposed framework operates on 2D MRI slices, standard anatomically plausible 2D geometric and intensity transformations were applied at the slice level, which is consistent with common practice in slice-based medical image classification.

### Proposed model architecture

The proposed dual-path deep learning framework (Fig. 1) concurrently performs brain tumor classification and content-based image retrieval (CBIR). The architecture comprises three main components: (i) a feature extraction backbone based on a modified GhostNetV3 architecture, (ii) a classification path for tumor type prediction, and (iii) a CBIR path to retrieve visually similar cases.

#### Feature extraction backbone

**Fig. 2:**
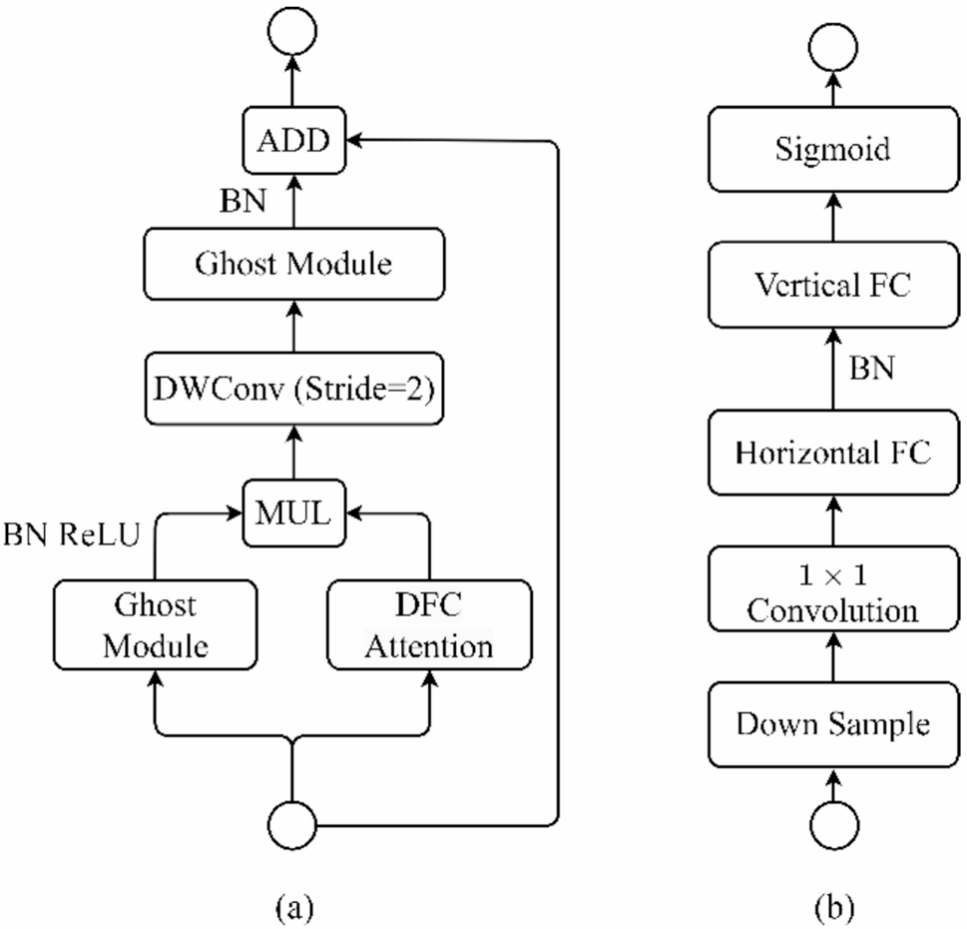
Architectures of (**a**) the modified GhostNetV3 bottleneck block and (**b**) DFC (Decoupled Fully Connected) attention mechanism

The proposed framework leverages a modified GhostNetV3 architecture as its core feature extraction backbone, depicted in Fig. 2. GhostNetV3 is selected due to its capability of capturing rich, high-level representations with minimal computational overhead [39]. To enhance its ability to extract tumor-specific features, we integrate two key modifications: deformable convolutions [40] within the Ghost modules and a Decoupled Fully Connected (DFC) attention mechanism [39]. These modifications enable the network to effectively capture both local and global features, crucial for accurate tumor characterization.

The feature extraction pipeline initiates by processing the input MRI image through an initial convolutional layer with a 3 × 3 kernel and stride of 2, followed by Batch Normalization (BN) and a Rectified Linear Unit (ReLU) activation. This initial stage extracts low-level features, reduces spatial dimensions, and prepares the input for subsequent processing within the modified GhostNetV3 bottleneck blocks (Fig. 2a).

**Fig. 3:**
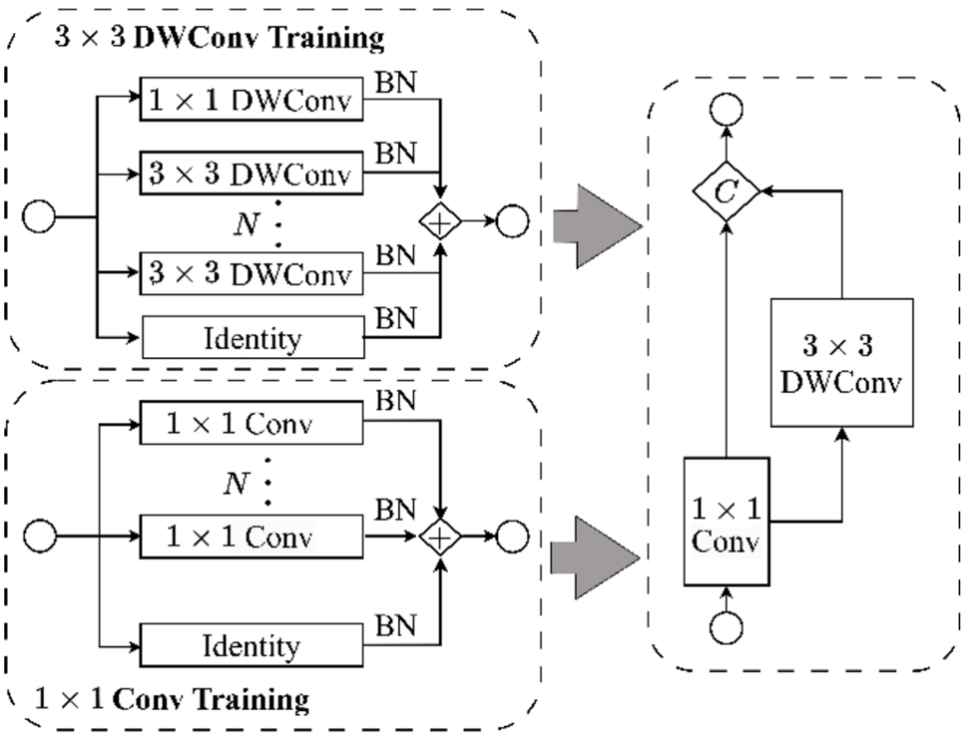
Detailed Ghost Module architecture in GhostNetV3, where $\:C$ denotes concatenation operation

Each bottleneck block within the architecture comprises two primary pathways: the Ghost module integrated with deformable convolutions (Fig. 3), and the Decoupled Fully Connected (DFC) attention mechanism (Fig. 2b). Within the Ghost module, standard convolutions are replaced by deformable convolutions to dynamically adapt the receptive field. The deformable convolution operation is defined as:

1 $$\:\mathrm{y}\left({\mathrm{p}}_{0}\right)=\sum\:_{{\mathrm{p}}_{\mathrm{n}}\in\:\mathrm{R}}\:\mathrm{w}\left({\mathrm{p}}_{\mathrm{n}}\right)\cdot\:\mathrm{x}\left({\mathrm{p}}_{0}+{\mathrm{p}}_{\mathrm{n}}+{\Delta\:}{\mathrm{p}}_{\mathrm{n}}\right),$$

where $\:{\mathrm{p}}_{0}$ denotes the output location, pₙ enumerates kernel positions in the receptive field $\:\mathcal{R}$, $\:\mathrm{w}\left({\mathrm{p}}_{n}\right)$ represents the convolutional weights, and Δpₙ denotes learned spatial offsets that deform the sampling grid. These offsets are generated by a parallel 3 × 3 convolutional layer with $2{K^2}$ output channels ($\:K\:=\:3$), applied to the input feature map and optimized end-to-end via backpropagation, enabling spatially adaptive receptive fields that better capture irregular tumor morphology.

Training of the Ghost modules further involves two parallel convolutional paths (Fig. 3): a 1 × 1 convolution path and a 3 × 3 depth-wise convolution path integrated with deformable convolutions. The outputs from these paths are concatenated to form a comprehensive feature representation.

To further enhance model efficiency and performance, GhostNetV3 utilizes advanced training strategies including convolutional re-parameterization and Knowledge Distillation (KD). Convolutional re-parameterization allows multiple parallel convolutional branches (e.g., 1 × 1 and 3 × 3 depth-wise convolutions, each with BatchNorm layers) to be trained independently. At inference, these branches are merged into a single equivalent convolutional layer via inverse re-parameterization, consolidating weights ($\:{\widehat{\mathbf{W}}}_{i}$) and biases ($\:{\widehat{\mathbf{b}}}_{i}$) from each branch (i):

2 $${{\bf{W}}_{{\rm{rep}}\>}} = \sum\nolimits_{\rm{i}} {{{{\rm{\hat W}}}_{\rm{i}}}} ,\,\,\,\,\,\,{{\bf{b}}_{{\rm{rep}}\>}} = \sum\nolimits_{\rm{i}} {{{{\rm{\hat b}}}_{\rm{i}}}}.$$

Knowledge distillation complements re-parameterization by transferring knowledge from a high-capacity, pre-trained teacher model to the compact GhostNetV3 student model through minimization of a combined loss function:

3 $$\eqalign{ {{\cal L}_{{\rm{total}}\>}} = & (1 - {\rm{\alpha }}){{\cal L}_{{\rm{ce}}}}\left( {\Gamma {\>_{\rm{s}}}\left( {\rm{x}} \right),{\rm{y}}} \right) \cr & + {\rm{\alpha }}{{\cal L}_{{\rm{kd}}}}\left( {{\Gamma _{\rm{s}}}\left( {\rm{x}} \right),{\Gamma _{\rm{t}}}\left( {\rm{x}} \right)} \right), \cr}$$

where $\:{\mathcal{L}}_{ce}$ denotes cross-entropy loss, $\:{\mathcal{L}}_{kd}$ denotes KD loss, $\:{{\Gamma\:}}_{s}\left(x\right)$ and $\:{{\Gamma\:}}_{t}\left(x\right)$ represent predictions from the student and teacher models respectively, $\rm{y}$ is the ground truth label, and $\:\alpha\:$ balances the contribution of each loss term.

In Eq. (3), $\:{{\Gamma\:}}_{s}\left(x\right)$ denotes the class probability predictions produced by the student model’s own classification head, implemented as a fully connected layer with softmax (described in Sect. “Dual-path diagnostic workflow”). This classification head and the feature extraction backbone are trained jointly in an end-to-end manner, and the predictions $\:{{\Gamma\:}}_{s}\left(x\right)$ are used directly to compute the classification loss and to guide feature learning through backpropagation.

The term $\:{{\Gamma\:}}_{t}\left(x\right)$ corresponds to the predictions of a separate, pre-trained, and fixed teacher model (ResNet50 pre-trained on ImageNet), which is used only during training to provide a soft supervisory signal via knowledge distillation. The teacher model is not used during inference. At test time, the student model’s classification head serves as the sole classifier for tumor prediction, while the extracted features are simultaneously reused for content-based image retrieval.

The KD loss typically employs Kullback-Leibler (KL) divergence, formulated as:

4 $$\eqalign{ {{\cal L}_{{\rm{kd}}}} = & {{\rm{\tau }}^2} \cr \cdot & {\rm{KL}}\left( {{\rm{softmax}}\left( {{{\Gamma {\>_{\rm{s}}}\left( {\rm{x}} \right)} \over {\rm{\tau }}}} \right),{\rm{softmax}}\left( {{{\Gamma {\>_{\rm{t}}}\left( {\rm{x}} \right)} \over {\rm{\tau }}}} \right)} \right), \cr}$$

where the temperature parameter $\:\tau\:$ controls the softness of probability distributions, facilitating smoother label representations and improving generalization.

Parallel to the Ghost module, the DFC attention mechanism (Fig. 2b) enhances feature representations by capturing long-range spatial dependencies. It initially downsamples feature maps via average pooling to reduce computational complexity, then employs a 1 × 1 convolution to effectively mix channel information. Subsequently, decoupled horizontal and vertical fully connected layers independently model spatial dependencies along respective axes, offering computational efficiency compared to traditional fully connected attention mechanisms. A sigmoid activation normalizes attention weights to the range [0,1], applied element-wise to original feature maps, thereby emphasizing diagnostically relevant regions and suppressing irrelevant information.

Outputs from the Ghost module and DFC attention pathways are combined via element-wise multiplication, effectively integrating spatially adaptive features from deformable convolutions with global context provided by attention. This fused representation subsequently undergoes dimensionality reduction through a stride2 depth-wise convolution (also integrated with deformable convolutions), followed by another Ghost module. A skip connection, implemented through element-wise addition, further refines the feature maps by merging fine-grained input details with high-level contextual information learned from the bottleneck block. The enriched feature maps from this backbone ultimately feed into downstream tumor classification and CBIR heads. The output of the final GhostNetV3 stage consists of 960 feature channels. After global average pooling, the final shared feature representation is a 960-dimensional vector, which serves as the input to both the classification head and the CBIR embedding space.

#### Dual-path diagnostic workflow

The extracted features from the modified GhostNetV3 backbone are processed by two parallel pathways: a classification path and a CBIR path (Fig. 1). This dual-path design enhances diagnostic confidence by providing both a direct tumor classification and visually similar reference cases, facilitating more informed clinical decision-making.

##### Classification path

The classification path predicts the tumor type (glioma, meningioma, or pituitary tumor). A fully connected layer followed by a softmax activation outputs a probability distribution over the three classes. The softmax activation is defined as:

5 $$\:{\widehat{\mathrm{y}}}_{\mathrm{i}}=\frac{\mathrm{exp}\left({\mathrm{z}}_{\mathrm{i}}\right)}{\sum\:_{\mathrm{k}=1}^{\mathrm{C}}\:\mathrm{e}\mathrm{x}\mathrm{p}\left({\mathrm{z}}_{\mathrm{k}}\right)},\:\:\:\:\:\:\:\:\:\mathrm{i}\in\:\left\{\mathrm{1,2},\dots\:,\mathrm{C}\right\},$$

Where $\:{z}_{i}$ represents the logits of class $\:i$, and $\:C\:=\:3$ denotes the number of tumor classes. This ensures the output probabilities are non-negative and sum to one, effectively quantifying classification confidence. Model training is guided by minimizing the categorical cross-entropy loss:

6 $${{\cal L}_{ce}} = - {1 \over {\rm{N}}}\sum {\>_{{\rm{j}} = 1}^{\rm{N}}} \>\sum {\>_{{\rm{i}} = 1}^{\rm{C}}} \>{{\rm{y}}_{{\rm{ij}}}}{\rm{log}}\left( {{{{\rm{\hat y}}}_{{\rm{ij}}}}} \right),$$

where $\:N$ is the number of training samples, $\:{y}_{ij}$ represents the true label (equal to 1 if sample $\:j$ belongs to class $\:i$, otherwise 0), and ${{{{\rm{\hat y}}}_{{\rm{ij}}}}}$ is the predicted probability for sample $\:j$ belonging to class $\:i$. To optimize this loss efficiently, the Adam optimizer is employed, leveraging its adaptive learning rate characteristics for accelerated convergence and improved model stability.  

##### CBIR path

The CBIR pathway provides interpretability by retrieving visually similar MRI scans from a pre-constructed database containing labeled images and their corresponding feature vectors. To prevent data leakage, the CBIR gallery is constructed exclusively from images belonging to the training split of each cross-validation fold. Query images are drawn strictly from the held-out test set, ensuring that no patient appears in both the gallery and the query set. This strict patient-level query–gallery separation ensures fair and unbiased retrieval evaluation. No independent held-out dataset is used; all reported results are obtained via 5-fold patient-level stratified cross-validation, where training, validation, and testing splits are redefined independently within each fold.

At inference time, a query MRI image’s extracted feature vector (x) is compared against the feature vectors stored in the database. To robustly measure image similarity, three complementary distance metrics are utilized:

- **Correlation Distance**, measuring linear dissimilarity based on the Pearson correlation coefficient:

7 $${{\rm{d}}_{{\rm{corr}}}}({\bf{x}},{\bf{y}}) = 1 - {{\sum {\>_{{\rm{i}} = 1}^{\rm{n}}} \>\>\left( {{{\rm{x}}_{\rm{i}}} - {\rm{\bar x}}} \right)\left( {{{\rm{y}}_{\rm{i}}} - {\rm{\bar y}}} \right)} \over {\sqrt {\sum {\>_{{\rm{i}} = 1}^{\rm{n}}} \>\>{{\left( {{{\rm{x}}_{\rm{i}}} - {\rm{\bar x}}} \right)}^2}} \sqrt {\sum {\>_{{\rm{i}} = 1}^{\rm{n}}} \>\>{{\left( {{{\rm{y}}_{\rm{i}}} - {\rm{\bar y}}} \right)}^2}} }},$$

where $\bar x$ and $\bar y$ are the means of vectors $\:\mathbf{x}$ and $\:\mathbf{y}$, respectively.

- **Standardized Euclidean Distance**, accounting for feature variance across the dataset:

8 $$\:{\mathrm{d}}_{\mathrm{seuclidean\:}}\left(\mathbf{x},\mathbf{y}\right)=\sqrt{\sum\:_{\mathrm{i}=1}^{\mathrm{n}}\:\:\frac{{\left({\mathrm{x}}_{\mathrm{i}}-{\mathrm{y}}_{\mathrm{i}}\right)}^{2}}{{\mathrm{s}}_{\mathrm{i}}^{2}}},$$

where $\:{s}_{i}$ represents the standard deviation of the $\:i$-th feature across all dataset images.

- **Spearman Rank Correlation Distance**, capturing monotonic relationships between ranked features:

9 $$\:{\uprho\:}\left(\mathbf{x},\mathbf{y}\right)=1-\frac{6\sum\:_{\mathrm{i}=1}^{\mathrm{n}}\:\:{\mathrm{d}}_{\mathrm{i}}^{2}}{\mathrm{n}\left({\mathrm{n}}^{2}-1\right)},$$

where $\:{d}_{i}$ denotes the rank difference between features $\:{x}_{i}$ and $\:{y}_{i}$.

These metrics collectively capture linear correlation, variance-aware Euclidean distance, and rank-based monotonic similarity, enabling robust retrieval by accounting for diverse aspects of visual similarity.

For each similarity metric $\:m$, the top 30 most similar images form an indexed set $\:{R}_{m}\left(\mathbf{x}\right)$. To further enhance retrieval robustness, a majority voting mechanism is applied across these sets. Each candidate image $\:j$ in the database receives a voting score $\:{V}_{j}\left(\mathbf{x}\right)$:

10 $$\:{\mathrm{V}}_{\mathrm{j}}\left(\mathbf{x}\right)=\sum\:_{\mathrm{m}\in\:\{\mathrm{C},\mathrm{S}\mathrm{E},\:\mathrm{S}\}}\:\mathbb{I}\left[\mathrm{j}\in\:{\mathrm{R}}_{\mathrm{m}}\left(\mathbf{x}\right)\right],$$

where $\:m$ denotes the employed distance metric (C: Correlation, SE: Standardized Euclidean, S: Spearman), and $\:\mathbb{I}$ is an indicator function returning 1 if image $\:j$ appears in the retrieval set $\:{R}_{m}\left(\mathbf{x}\right)$, otherwise 0. The top 10 images receiving the highest votes constitute the final set of visual references presented to clinicians.

By integrating multiple similarity measures through majority voting, the proposed CBIR system effectively reduces biases inherent to individual metrics, significantly enhancing retrieval accuracy. Moreover, providing diverse yet relevant reference images substantially improves interpretability, assisting clinicians in comparative analysis and informed diagnostic decision-making.

### Training protocol and evaluation

To ensure reproducibility, the complete training and evaluation workflow is summarized in Algorithm 1. We employ a 5-fold patient-level stratified cross-validation protocol, ensuring that all MRI slices belonging to the same patient are assigned exclusively to a single fold, thereby preventing data leakage caused by inter-slice correlations.

**Algorithm 1:**
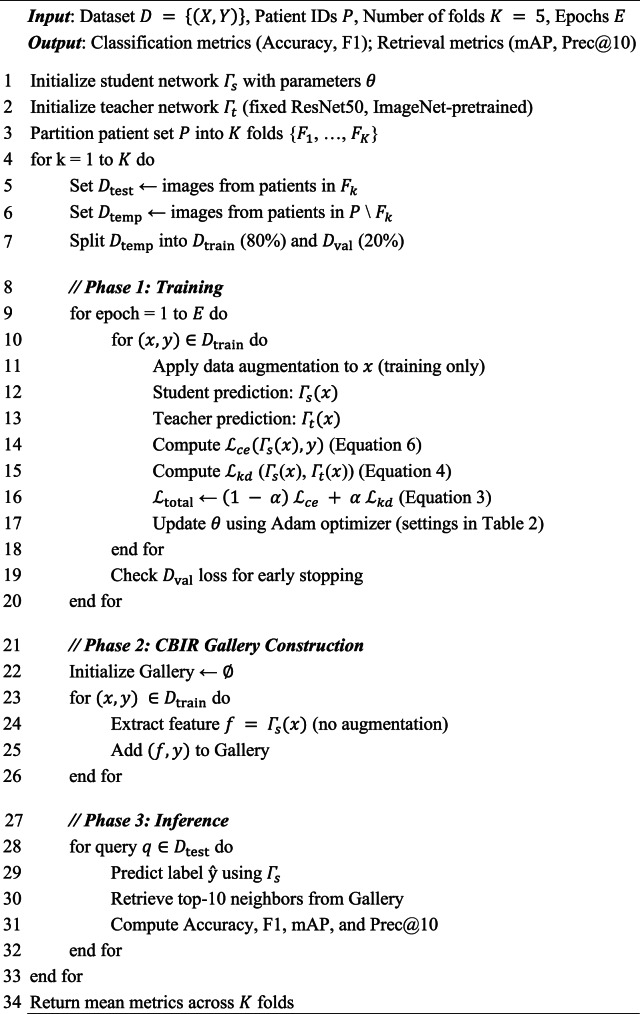
Training and evaluation

## Numerical results and discussion

This section provides a comprehensive evaluation of the proposed dual-path deep learning framework for brain tumor diagnosis using the publicly available CE-MRI dataset. To thoroughly assess performance, we first evaluate the tumor classification and content-based image retrieval (CBIR) components individually, followed by a joint analysis demonstrating their synergistic integration. For both classification and CBIR evaluation, a 5-fold patient-level stratified cross-validation protocol was applied to ensure robust performance estimation and prevent inter-patient data leakage. All reported results were obtained under identical training and evaluation settings, using the hyperparameter configuration detailed in Table 2.

### Classification results

The classification model is trained using the Adam optimizer (learning rate: 0.0001), categorical cross-entropy loss, and ReLU activation function. Early stopping, activated after 5 epochs without improvement in validation loss, prevents overfitting and ensures stable, generalizable performance. The classification results are rigorously evaluated using 5-fold stratified cross-validation. Accuracy, Precision, Recall, and F1-score are computed to comprehensively assess classification performance, defined as follows:

11 $$\:\mathrm{Accuracy}=\frac{\mathrm{T}\mathrm{P}+\mathrm{T}\mathrm{N}}{\mathrm{T}\mathrm{P}+\mathrm{T}\mathrm{N}+\mathrm{F}\mathrm{P}+\mathrm{F}\mathrm{N}},$$

12 $$\:\mathrm{Precision}=\frac{\mathrm{T}\mathrm{P}}{\mathrm{T}\mathrm{P}+\mathrm{F}\mathrm{P}},$$

13 $$\:\mathrm{Recall}=\frac{\mathrm{T}\mathrm{P}}{\mathrm{T}\mathrm{P}+\mathrm{F}\mathrm{N}},$$

14 $${\rm{F1 - Score}} = 2 \times {{{\rm{Precision}} \times {\rm{Recall}}} \over {{\rm{Precision + Recall}}}},$$

where$\:\:\mathrm{T}\mathrm{P},\:\mathrm{T}\mathrm{N},\:\mathrm{F}\mathrm{P}$, and $\:\mathrm{F}\mathrm{N}$ represent True Positives, True Negatives, False Positives, and False Negatives, respectively. For multi-class classification, these metrics are calculated per class and then macro-averaged to provide an overall, balanced performance measure.

**Table 2:** Hyperparameter configuration

Hyperparameter	Value
Learning rate	0.0001
Batch size	32
Weight decay	0.0001
Dropout rate	0.3
Epochs	40
Deformable conv offset groups	4
DFC attention reduction ratio	8
Knowledge distillation (α)	0.7
Temperature parameter (τ)	4
Data augmentation probability	0.5

**Fig. 4:**
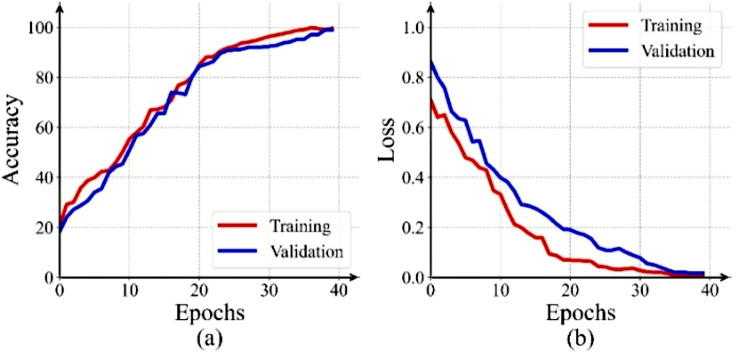
Training and validation performance curves: (**a**) Accuracy, (**b**) Loss

The training dynamics of the proposed classification model are illustrated through accuracy and loss curves in Fig. 4. The consistent decrease in loss and increase in accuracy across both training and validation datasets indicate effective learning and convergence within 40 epochs. The close alignment between the training and validation curves further suggests strong generalization performance. The plateauing of validation metrics before the 40-epoch limit demonstrates the effectiveness of early stopping.

Table 3 summarizes the overall classification performance, reporting mean values and standard deviations computed across the 5-fold cross-validation. The proposed model achieves remarkable classification accuracy (99.71%), precision (99.66%), recall (99.62%), and F1-score (99.64%), underscoring its effectiveness in accurately distinguishing between brain tumor classes.

**Table 3:** Overall classification performance

Metric	(Mean ± STD)
Accuracy	0.9971 ± 0.004
Precision	0.9966 ± 0.006
Recall	0.9962 ± 0.008
F1-Score	0.9964 ± 0.004

**Fig. 5:**
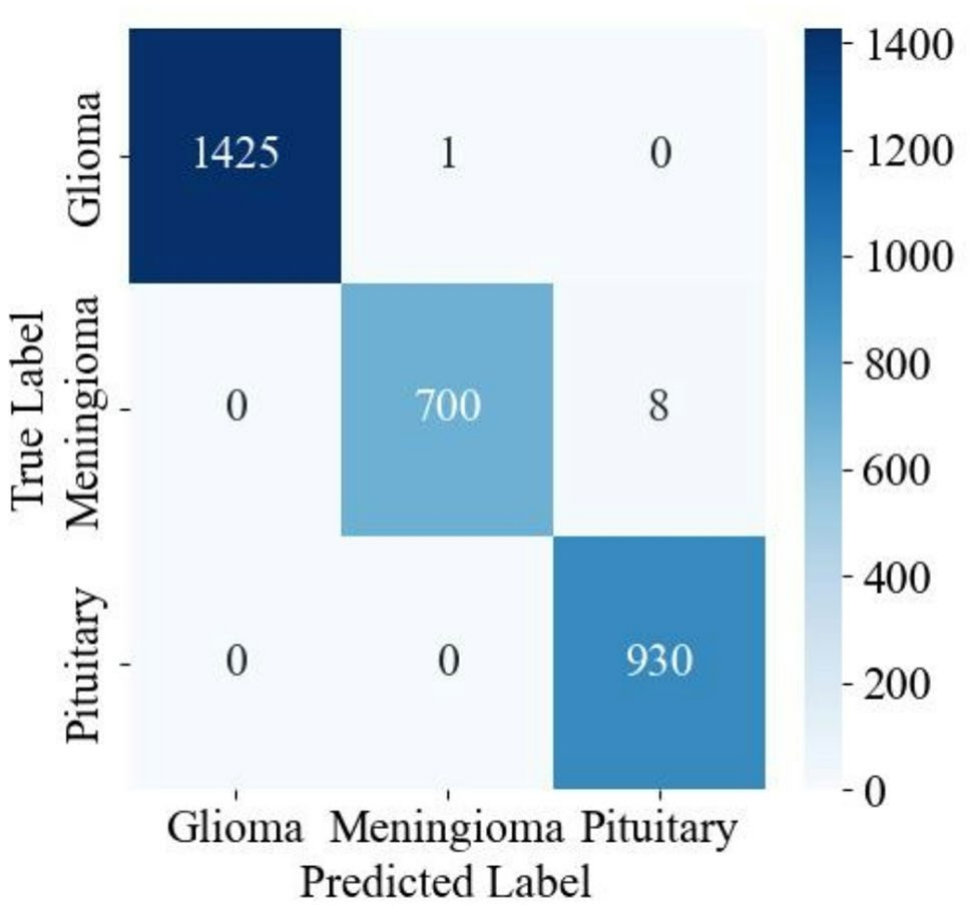
Confusion matrix of the proposed classification model

The class-specific classification performance, presented in Table 4, highlights consistently high precision, recall, and F1-score across all tumor classes (glioma, meningioma, and pituitary), with each metric exceeding 98.9%. This balanced and uniformly strong performance underscores the model’s robustness and capability to accurately distinguish among different tumor types. To further visualize this per-class performance, Fig. 5 presents the confusion matrix. The high diagonal values clearly reflect the accurate classification within each tumor category, while the minimal off-diagonal values indicate negligible inter-class confusion, further demonstrating the classifier’s effectiveness in differentiating between tumor types.

**Fig. 6:**
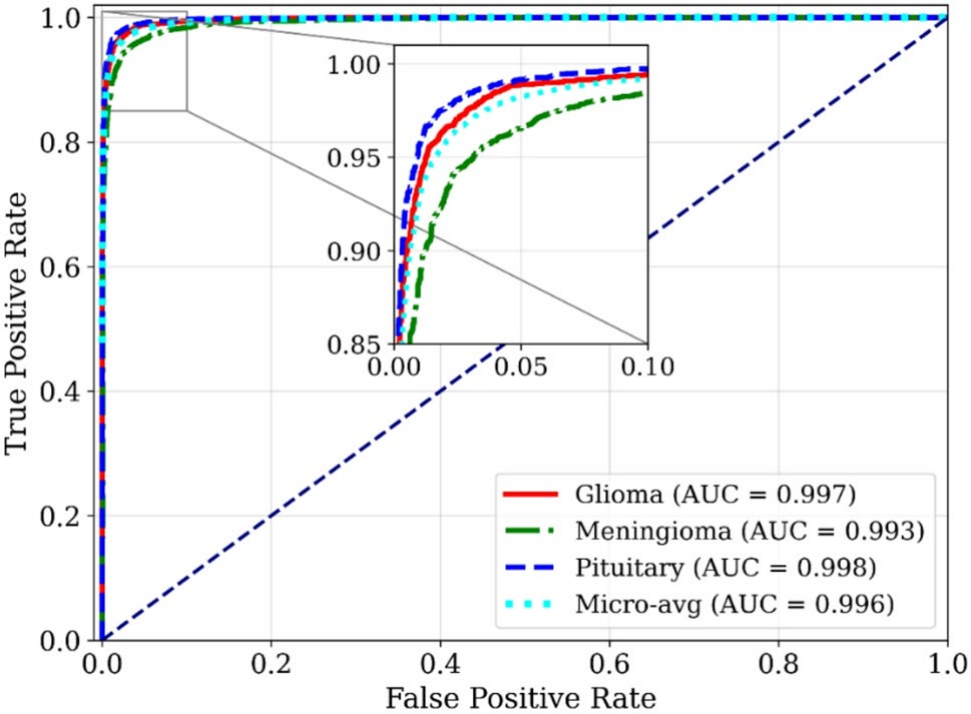
Receiver operating characteristic (ROC) curves and corresponding AUC values for each tumor class and the micro-average. The inset highlights performance at low false-positive rates

The Receiver Operating Characteristic (ROC) curves in Fig. 6 further validate the discriminatory ability of the proposed classifier. The model achieves near-perfect Area Under Curve (AUC) scores of approximately 1.00 for glioma and pituitary tumors, and approximately 0.99 for meningioma, emphasizing strong differentiation across tumor classes. Furthermore, the near-perfect micro-averaged AUC (≈ 1) reinforces the classifier’s robustness in handling class imbalance and its consistent reliability across diverse testing scenarios, demonstrating exceptional discriminatory performance.

**Table 4:** Class-Specific classification performance

Class	Precision	Recall	F1-Score
Glioma	1.0000	0.9993	0.9997
Meningioma	0.9986	0.9894	0.9939
Pituitary	0.9913	1.0000	0.9956

**Fig. 7:**
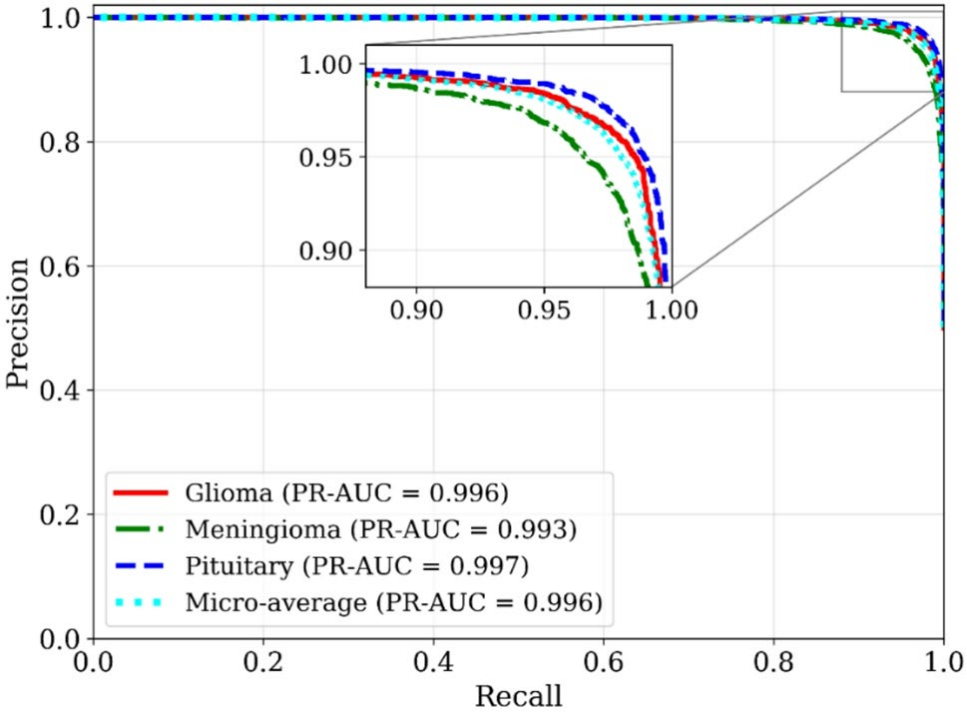
Precision–Recall (PR) curves for glioma, meningioma, and pituitary tumor classes, along with the micro-average. The inset highlights performance in the high-recall region

To further complement the ROC analysis, Fig. 7 presents the Precision–Recall (PR) curves and corresponding PR-AUC values for each tumor class and the micro-average. Unlike ROC curves, PR curves emphasize performance on the positive class and are particularly informative for imbalanced medical datasets. The proposed framework achieves consistently high PR-AUC values (≥ 0.993) across all tumor categories, aligning closely with the strong precision, recall, and F1-scores reported in Tables 3 and 4. These results further confirm the robustness and reliability of the classifier across clinically relevant operating points.

### Comparison with state-of-the-art classification models

To more rigorously validate the classification efficacy of our proposed dual-path framework, we benchmarked its classification performance against a set of widely adopted deep learning architectures representing both CNN and Transformer families: VGG16 [42], ResNet50 [43], DenseNet121 [44], and Vision Transformer (ViT-B/16) [45]. All baseline models were trained and evaluated under the same experimental protocol used for our framework to ensure a fair comparison. These architectures were selected to represent standard high-capacity CNN and Transformer benchmarks against which the parameter-efficient design of GhostNetV3 can be meaningfully evaluated.

We used a 5-fold patient-level stratified cross-validation (stratification at patient level to preserve class balance across folds) and report macro-averaged metrics (Accuracy, Precision, Recall, F1-Score) computed across folds. To avoid data leakage, all image preprocessing and patient-wise fold assignment were performed prior to any augmentation; data augmentation was applied only to the training split within each fold. Baseline models were initialized with ImageNet pre-trained weights and fine-tuned using the hyperparameters listed in Table 2 (learning rate = 1e-4, batch size = 32, up to 40 epochs with early stopping after 5 epochs of no validation improvement). Input resolution, optimizer, learning-rate schedule, and augmentation protocol were kept consistent across models.

Table 5 summarizes the comparative classification results (mean ± standard deviation over 5 patient-level folds) and model parameter counts. We additionally evaluated statistical significance of the accuracy differences across folds using a paired Wilcoxon signed-rank test (paired by fold). The proposed framework’s accuracy improvement over the strongest baseline (DenseNet121) is statistically significant (Wilcoxon *p* < 0.05).

**Table 5:** Comparative classification performance against SOTA baselines (Mean ± STD over 5 folds)

Model	Accuracy (%)	Precision (%)	Recall (%)	F1-Score (%)	Params (M)
VGG16 [42]	97.42 ± 0.35	97.38 ± 0.41	97.40 ± 0.38	97.39 ± 0.39	138.4
ResNet50 [43]	98.15 ± 0.28	98.12 ± 0.30	98.10 ± 0.32	98.11 ± 0.29	25.6
Vision Transformer (ViT-B/16) [45]	98.24 ± 0.30	98.21 ± 0.33	98.25 ± 0.29	98.23 ± 0.31	86.6
DenseNet121 [44]	98.63 ± 0.21	98.60 ± 0.22	98.58 ± 0.24	98.59 ± 0.22	8.0
Proposed	99.71 ± 0.40	99.66 ± 0.61	99.62 ± 0.79	99.64 ± 0.40	~ 5.2

As shown in Table 5, our proposed framework attains the highest macro-averaged metrics, achieving a mean accuracy of 99.71%, which exceeds DenseNet121 by approximately 1.08% points. Importantly, our model delivers this performance with notably fewer parameters (~ 5.2 M) than ResNet50, ViT, and VGG16, improving parameter efficiency and reducing memory/compute requirements—an important consideration for potential clinical deployment on resource-limited hardware.

While Transformer-based ViT achieves competitive accuracy, it exhibits a substantially larger parameter count and non-negligible variance across folds, consistent with the known sensitivity of transformer models to dataset size and the lack of local inductive biases that are often helpful on smaller medical imaging datasets. The observed performance advantage of our method is likely due to the combination of a parameter-efficient GhostNetV3 backbone, which benefits from Ghost modules and re-parameterization strategies, together with deformable convolutions that allow adaptive receptive fields matching irregular tumor shapes and the focused contextual adaptation provided by the DFC attention mechanism. We note that definitive attribution of performance gains to individual components is provided by the ablation study in Sect. “Ablation study” (Ablation Study), which isolates deformable convolutions, the DFC attention, and the dual-path design.

### Ablation study

To isolate and quantify the individual contributions of the key architectural components—namely, the deformable convolutions, the Decoupled Fully Connected (DFC) attention mechanism, and the dual-path joint optimization strategy—we conducted a comprehensive ablation study. This analysis aims to disentangle the effects of each component and to substantiate the design choices underlying the proposed framework. All ablation experiments were performed using the identical 5-fold patient-level stratified cross-validation protocol described in Sect. "Comparison with state-of-the-art classificatio nmodels". To ensure fairness and reproducibility, all training conditions were kept identical across variants, including input resolution, data preprocessing, augmentation strategy (applied exclusively to training splits), optimizer settings, learning rate, number of epochs, and early stopping criteria. Each variant employs the same GhostNetV3 backbone initialization and training schedule, differing only in the architectural component under investigation.

Five experimental configurations were defined to systematically assess component contributions. The baseline model, A1 (GhostNetV3), employs the standard backbone trained exclusively for classification, without deformable convolutions, DFC attention, or the CBIR pathway. To isolate individual architectural enhancements, A2 integrates deformable convolutions into the Ghost modules while keeping the DFC attention mechanism and CBIR branch disabled, whereas A3 incorporates the DFC attention mechanism while retaining standard convolutions. The combined influence of these modules is evaluated in A4 (Single-Path Classification), where both deformable convolutions and DFC attention are enabled within a classification-only framework. Finally, A5 represents the full proposed dual-path framework, which integrates all architectural enhancements with joint optimization for simultaneous tumor classification and content-based image retrieval. Table 6 summarizes the impact of these architectural components on classification performance, retrieval effectiveness, and parameter efficiency. Reported values correspond to the mean ± standard deviation across five patient-level folds, reflecting inter-patient variability rather than slice-level fluctuations.

**Table 6:** Ablation study of key architectural components (Mean ± STD over 5 folds)

Configuration	Accuracy (%)	F1-Score (%)	Retrieval mAP (%)	Prec@10 (%)	Params (M)
A1: Baseline (GhostNetV3)	98.15 ± 0.45	98.11 ± 0.46	95.23 ± 0.25	99.12 ± 0.15	4.7
A2: A1 + Deformable Convs	99.02 ± 0.42	98.98 ± 0.43	96.45 ± 0.21	99.45 ± 0.12	5.0
A3: A1 + DFC Attention	98.87 ± 0.44	98.84 ± 0.45	96.18 ± 0.23	99.38 ± 0.13	4.9
A4: A1 + DC + DFC (Single-Path)	99.48 ± 0.42	99.45 ± 0.44	97.12 ± 0.20	99.68 ± 0.09	5.2
A5: Full Dual-Path (Proposed)	99.71 ± 0.40	99.64 ± 0.40	97.74 ± 0.19	99.78 ± 0.03	5.2

Statistical analysis using a paired Wilcoxon signed-rank test on fold-wise accuracy indicates that the full dual-path model achieves significantly higher performance compared to the ablated variants (*p* < 0.05).

Several observations emerge from the ablation results. First, the baseline GhostNetV3 backbone (A1) achieves competitive classification performance (98.15% accuracy), confirming its suitability as a lightweight feature extractor for brain tumor MRI. However, it underperforms the full model by more than 1.5% points, highlighting the benefit of the proposed architectural enhancements. Second, integrating deformable convolutions (A2) leads to a substantial improvement in both classification accuracy (+ 0.87%) and retrieval performance (+ 1.22% mAP). This finding supports the importance of adaptive receptive fields for modeling the irregular shapes and heterogeneous boundaries characteristic of brain tumors, which are not optimally captured by fixed-grid convolutions. Third, incorporating the DFC attention mechanism alone (A3) also improves performance relative to the baseline, although to a slightly lesser extent. By modeling long-range spatial dependencies, DFC attention enables the network to emphasize diagnostically relevant regions while suppressing background structures. Fourth, combining deformable convolutions and DFC attention in a single-path classification setting (A4) yields a larger performance gain than either component alone, demonstrating their complementary nature. Deformable convolutions primarily enhance local spatial adaptability, whereas DFC attention contributes global contextual awareness.

Finally, the full dual-path framework (A5) achieves the highest performance across all evaluated metrics, including both classification and retrieval. The performance gap between A4 and A5 indicates that joint optimization of classification and CBIR provides an additional regularization effect, encouraging the backbone to learn feature representations that are simultaneously discriminative and semantically consistent. Importantly, this multi-task benefit is achieved without additional inference-time parameters compared to the single-path configuration, reinforcing the efficiency of the proposed design. This synergy is further reflected in the high Classification–Retrieval Agreement Score (CRAS) reported in Sect. "Joint evaluation". Overall, the ablation study confirms that the gains of the proposed framework arise from the synergistic integration of deformable convolutions, DFC attention, and dual-path learning, rather than from any single component in isolation.

### Retrieval results

The performance of the CBIR system is assessed using the majority voting scheme evaluated through 5-fold stratified cross-validation. To comprehensively evaluate retrieval effectiveness, three standard metrics are reported: Precision@K (Prec@K), Average Precision (AP), and mean Average Precision (mAP). For a given query image $\:q$, Prec@K measures the proportion of relevant images within the top-$\:K$ retrieved results and is defined as:

15 $$\:\mathrm{P}\mathrm{r}\mathrm{e}\mathrm{c}@\text{}\mathrm{K}\left(\mathrm{q}\right)=\frac{1}{\mathrm{K}}\sum\:_{\mathrm{r}\in\:{\mathrm{R}}_{\mathrm{q}}^{\mathrm{K}}}\:\mathrm{r}\mathrm{e}\mathrm{l}\left(\mathrm{q},\mathrm{r}\right),$$

where $\:{R}_{q}^{K}$ denotes the set of top-$\:K$ retrieved images, and rel$\:(q,\:r)\:$is an indicator function reflecting image relevance (1 if relevant, 0 otherwise). Similarly, Average Precision (AP) incorporates ranking order by computing the average of Prec@K at positions of relevant retrieved images:

16 $$\:\mathrm{A}\mathrm{P}\left(\mathrm{q}\right)=\frac{\sum\:_{\mathrm{k}=1}^{\mathrm{K}}\:\:\mathrm{P}\mathrm{r}\mathrm{e}\mathrm{c}@\mathrm{k}\left(\mathrm{q}\right)\:\times\:\:\mathrm{r}\mathrm{e}\mathrm{l}\left(\mathrm{q},{\mathrm{r}}_{\mathrm{k}}\right)}{\sum\:_{\mathrm{r}\in\:{\mathrm{R}}_{\mathrm{q}}^{\mathrm{K}}}\:\:\mathrm{r}\mathrm{e}\mathrm{l}(\mathrm{q},\mathrm{r})},$$

where$\:{\:r}_{k}$ represents the $\:k$-th retrieved image. The mAP metric then averages AP across all query images, providing an overall measure of retrieval effectiveness:

17 $$\:\mathrm{m}\mathrm{A}\mathrm{P}=\frac{1}{\left|\mathrm{Q}\right|}\sum\:_{\mathrm{q}\in\:\mathrm{Q}}\:\mathrm{A}\mathrm{P}\left(\mathrm{q}\right),$$

with $\:\left|Q\right|\:$denoting the total number of query images. The majority voting retrieval scheme is benchmarked against individual similarity metrics, with results summarized in Table 7. Among individual metrics, Spearman rank correlation achieves the highest retrieval performance, with an mAP of 97.01% ± 0.11% and Prec@10 of 99.56% ± 0.01%. However, our proposed voting scheme significantly outperforms all individual metrics, achieving an mAP of 97.74% ± 0.19% and Prec@10 of 99.78% ± 0.03%. This notable performance boost highlights the effectiveness of combining multiple similarity metrics through majority voting, leading to reduced metric biases and more accurate retrieval results.

**Table 7:** Retrieval performance (mAP and Prec@10) with Mean ± Standard deviation over 5-fold cross-validation

Retrieval Method	mAP (%)	Prec@10 (%)
Correlation	96.38 ± 0.18	99.54 ± 0.00
Standardized Euclidean	95.09 ± 0.04	99.49 ± 0.01
Spearman Rank	97.01 ± 0.11	99.56 ± 0.01
Voting Scheme	**97.74 ± 0.19**	**99.78 ± 0.03**

Retrieval performance across individual cross-validation folds is detailed in Table 8. The results highlight stable and consistently high performance for all folds. The narrow range of performance metrics across folds illustrates the robustness and strong generalizability of the proposed retrieval method. Furthermore, the high average mAP (97.74% ± 0.19%) and Prec@10 (99.78% ± 0.03%) underscore the effectiveness and reliability of the CBIR system.

A per-tumor type breakdown of retrieval performance is provided in Table 9, highlighting consistent accuracy across different tumor categories. Glioma demonstrates the highest mAP (99.77% ± 0.20%), while Meningioma achieves the highest Prec@10 (99.85% ± 0.03%). These high scores, consistently above 95% for mAP and 99% for Prec@10, affirm the CBIR system’s effectiveness in retrieving relevant, diagnostically similar images across diverse tumor types.

**Table 8:** Retrieval performance across five folds

Fold	mAP (%)	Prec@10 (%)
Fold 1	99.756	100.0
Fold 2	98.572	99.836
Fold 3	94.845	99.177
Fold 4	97.459	99.929
Fold 5	98.091	99.946
Mean ± STD	97.74 ± 0.19	99.78 ± 0.03

To evaluate the retrieval performance of our CBIR scheme in the context of recent literature, we compared its metrics against those reported by recently published methods in medical image retrieval as shown in Table 10. Our proposed framework, leveraging a modified GhostNetV3 backbone with deformable convolutions, DFC attention, and a majority voting scheme, achieves a mAP of 97.74% and a Prec@10 of 99.78% on the Cheng dataset, based on 5-fold patient-level cross-validation.

**Table 9:** Tumor-Specific retrieval performance (Mean ± STD)

Tumor Type	mAP (%)	Prec@10 (%)
Glioma	99.77 ± 0.20	99.71 ± 0.04
Meningioma	95.24 ± 0.18	99.85 ± 0.03
Pituitary	98.23 ± 0.18	99.77 ± 0.03

These performance metrics compare favorably with recently published results. For example, our achieved mAP is 2.06% points higher than the value reported for the hybrid optimization approach of Shetty et al. [31] (95.68%) and 3.5% points higher than that reported for the slice embedding technique of Tomoshige et al. [30] (94.24%). Likewise, our Prec@10 score indicates extremely high relevance among the top-ranked retrieved images and is numerically higher than the 98.17% reported for the fusion-based method of Iqbal et al. [28]. Because prior publications typically report only a single aggregated mean value without fold-wise distributions, formal paired hypothesis testing against those benchmarks is not applicable. Instead, we report descriptive comparisons: our 5-fold mAP and Prec@10 exceed the cited works by the margins shown in Table 10, indicating a clear performance advantage. For internal comparisons where fold-wise data is available (Table 6), we apply the paired Wilcoxon signed-rank test.

**Table 10:** Performance comparison of the proposed CBIR against recently published medical CBIR methods. Performance values for existing methods are cited from their respective original publications

Method (Reference)	mAP (%)	Prec@10 (%)
Iqbal et al. (2023) [28]	96.32	98.17
Singh et al. (2023) [27]	96.33	97.07
Rashad et al. (2023) [46]	95.52	94.97
Shetty et al. (2024) [31]	95.68	98.68
Tomoshige et al. (2025) [30]	94.24	97.65
**Proposed**	**97.74**	**99.78**

### Joint evaluation

The integration of classification and CBIR components aims to leverage their complementary strengths, enhancing diagnostic confidence by providing both predictive labels and visually similar reference cases. To quantitatively assess this synergy, we introduce and evaluate a novel metric called the Classification-Retrieval Agreement Score (CRAS). The CRAS measures how closely the classifier’s predicted label aligns with the labels of the top-$\:K$ retrieved images, assigning greater weight to images ranked higher due to their increased relevance:

18 $$\:\mathrm{C}\mathrm{R}\mathrm{A}\mathrm{S}\left(\mathrm{q}\right)=\frac{1}{\mathrm{W}}\sum\:_{\mathrm{r}\in\:{\mathrm{R}}_{\mathrm{q}}^{\mathrm{K}}}\:\frac{\mathbb{I}\left[\mathrm{C}\right(\mathrm{q})=\mathrm{G}\mathrm{T}(\mathrm{r}\left)\right]}{\mathrm{r}\mathrm{a}\mathrm{n}\mathrm{k}\left(\mathrm{r}\right)},$$

where $\:{R}_{q}^{K}$ denotes the top $\:K$ retrieved images for query $\:q$, $\:C\left(q\right)$ is the classifier’s predicted label, $\:\mathrm{G}\mathrm{T}\left(r\right)$ is the ground truth label of the retrieved image $\:r$, and $\:\mathbb{I}\left[C\right(q)=\mathrm{G}\mathrm{T}(r\left)\right]$ is an indicator function equal to 1 if labels match and 0 otherwise. The normalization term $\:W$ ensures CRAS scores remain within [0,1]:

19 $$\:\mathrm{W}=\sum\:_{\mathrm{r}\in\:{\mathrm{R}}_{\mathrm{q}}^{\mathrm{K}}}\:\frac{1}{\mathrm{r}\mathrm{a}\mathrm{n}\mathrm{k}\left(\mathrm{r}\right)},$$

Higher CRAS scores indicate stronger alignment between classifier predictions and retrieved image labels, thus reinforcing diagnostic certainty. A low CRAS suggests potential discrepancies that warrant further investigation.

The robustness and generalizability of this integrated diagnostic approach are assessed using 5-fold cross-validation, with the CRAS scores for each fold summarized in Table 11. The consistently high mean CRAS of 96.08% ± 3.43% clearly illustrates the strong agreement between classifier predictions and retrieved visual references, emphasizing the complementary role of CBIR in supporting classifier decisions.

**Table 11:** Average CRAS across five folds validation

Fold	CRAS
Fold 1	0.9865
Fold 2	0.9753
Fold 3	0.9055
Fold 4	0.9989
Fold 5	0.9380
Mean ± STD	0.9608 ± 0.0343

**Fig. 8:**
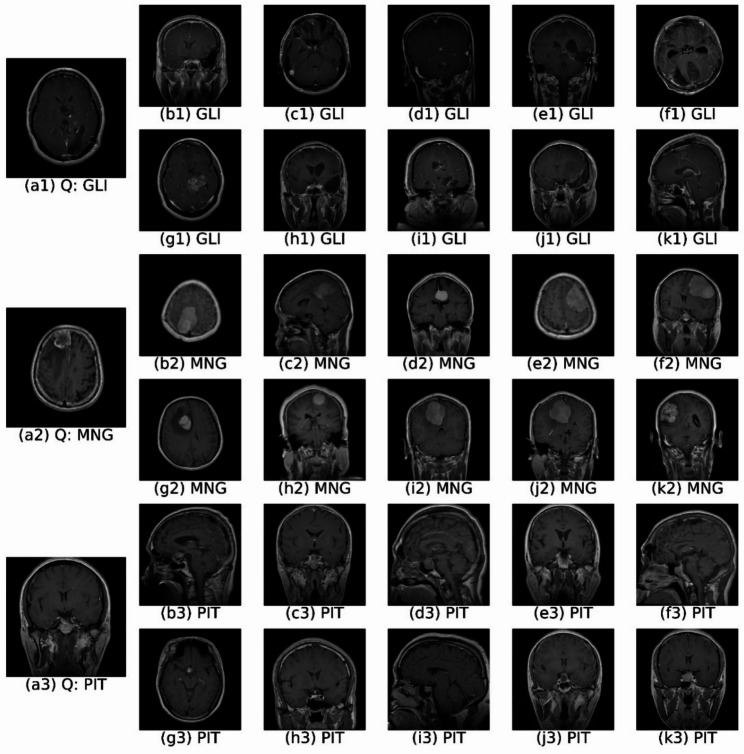
Top-10 retrieval results for query images of (a1) glioma, (a2) meningioma, and (a3) pituitary tumors showing perfect retrieval alignment (CRAS = 1.0). Clinically, consistent retrieval of same-class cases reinforces the automated classification and supports diagnostic confidence

**Fig. 9:**
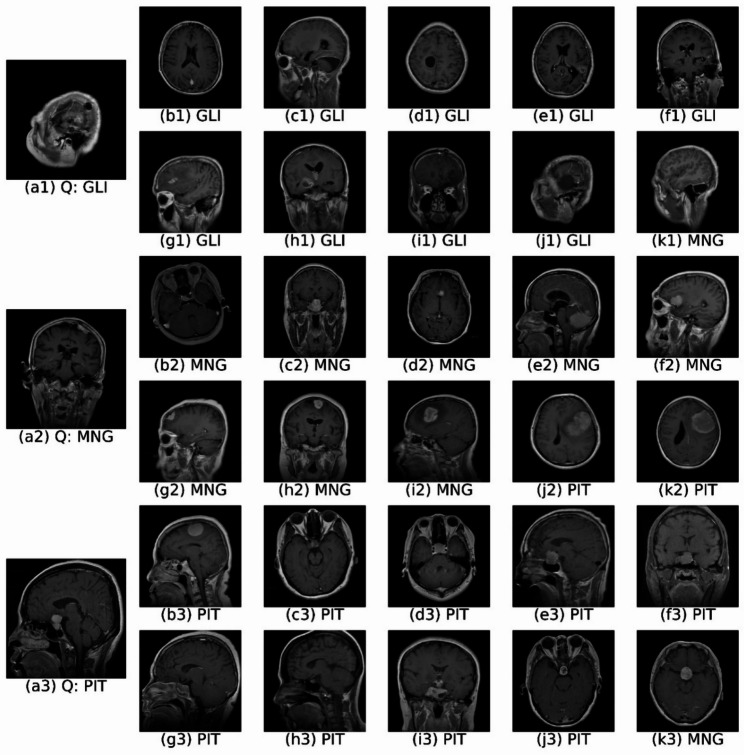
Top-10 retrieval results for query images of (a1) glioma, (a2) meningioma, and (a3) pituitary tumors with partial retrieval mismatches. The presence of visually similar cases from different classes highlights morphological overlaps and provides useful context for differential diagnosis

To qualitatively demonstrate how retrieved images can reinforce diagnostic predictions, Figs. 8 and 9 present retrieval examples illustrating both high-confidence and challenging scenarios.

Figure 8 highlights examples where retrieval results perfectly align with classifier predictions (CRAS = 1.0). For instance, the glioma query (Fig. 8 (a1), confidence 0.9831) retrieves ten glioma images, yielding AP@10 and CRAS scores of 1.0. Similar perfect alignment is observed for meningioma (confidence 0.9901) and pituitary (confidence 0.9886) queries. These cases illustrate clearly how CBIR can strengthen classifier predictions, particularly in high-confidence situations.

Conversely, Fig. 9 presents cases where some retrieval errors occur, illustrating scenarios of partial disagreement between classification and retrieval. In the glioma case (Fig. 9 (a1), classified with confidence 0.9726), nine out of ten retrieved images correctly match the glioma class, but the tenth image (k1) is a meningioma case, resulting in an AP@10 of 0.9 and a CRAS of 0.9659. This discrepancy highlights how visually similar cases from other diagnostic categories can appear among retrieval results, offering valuable additional context and prompting more careful review.

Similarly, the meningioma case (Fig. 9 (a2), confidence 0.9730) retrieves two pituitary tumor images (j2 and k2), leading to an AP@10 of 0.8 and a CRAS of 0.9279, indicating greater variation within the retrieval set. Finally, the pituitary case (Fig. 9 (a3), confidence 0.9813) retrieves one meningioma image (k3), yielding an AP@10 of 0.9 and CRAS of 0.9659. This exemplifies how even a single retrieved image with a different label can reveal subtle variations within the dataset and offer valuable insights, potentially prompting further investigation.

Collectively, these qualitative examples demonstrate that imperfect retrieval results can still contribute meaningful contextual information. They highlight subtle dataset variations, encourage deeper investigation, and ultimately promote a more nuanced, comprehensive diagnostic evaluation, reinforcing the practical value of integrating CBIR into clinical decision-making processes.

Building on the qualitative retrieval examples shown in Fig. 9, a deeper failure analysis reveals that residual errors arise primarily in cases exhibiting strong visual overlap between tumor types. Misclassifications and partial retrieval mismatches are most frequently observed between glioma and meningioma or between meningioma and pituitary tumors, which can share similar intensity patterns, anatomical locations, or boundary characteristics in contrast-enhanced MRI slices.

From a diagnostic reliability perspective, these failure modes reflect inherent image-level ambiguity rather than model instability. In such cases, the classifier may assign a confident prediction while the retrieval branch surfaces a small number of visually similar cases from other classes, resulting in reduced CRAS values. Importantly, this behavior exposes diagnostic uncertainty instead of concealing it, encouraging closer clinical scrutiny rather than blind reliance on automated labels. Consequently, the framework functions as a decision-support tool that highlights ambiguous cases where expert interpretation and complementary clinical context remain essential.

## Discussion

In this section, we present an in-depth interpretation of the experimental results, benchmark the proposed framework against existing state-of-the-art methods, and discuss the key limitations of the study.

### Interpretation of results

The experimental results demonstrate that the proposed dual-path framework achieves consistently high performance across both tumor classification and content-based image retrieval tasks. Beyond high predictive metrics, the results indicate that the modified GhostNetV3 backbone—enhanced with deformable convolutions and Decoupled Fully Connected (DFC) attention—learns feature representations that are both discriminative and semantically meaningful. As demonstrated in the ablation study (Sect. "Ablation study", Table 6), deformable convolutions play a key role in modeling irregular tumor boundaries, while DFC attention improves the network’s focus on diagnostically relevant regions. The stability of performance across patient-level cross-validation folds further suggests robust learning behavior and reduced susceptibility to overfitting, despite the limited dataset size.

The retrieval results complement the classification findings by providing visually and semantically similar reference cases with high relevance, as reflected by strong mAP and Prec@10 scores (Tables 7 and 9). The consistently high Classification–Retrieval Agreement Score (Table 11) confirms strong alignment between classifier predictions and retrieved cases, reinforcing the complementary nature of the two tasks. This dual confirmation—through both predictive confidence and case-based visual evidence—closely resembles clinical reasoning, where radiologists often cross-reference diagnostic hypotheses with similar historical cases to increase confidence and reduce uncertainty.

In addition to retrieval-based interpretability, the DFC attention mechanism contributes to model transparency by modulating feature responses along spatial dimensions. By independently modeling horizontal and vertical contextual dependencies, DFC attention selectively amplifies anatomically meaningful tumor patterns while suppressing background structures. This selective emphasis enhances the semantic relevance of both classification predictions and retrieved reference cases. This behavior increases clinician trust by ensuring that both predicted labels and retrieved reference cases are driven by tumor-relevant structures rather than spurious background cues. As a result, DFC attention provides an intrinsic form of interpretability embedded directly within the feature-learning process, rather than relying exclusively on post-hoc explanation techniques.

### Comparison with existing methods

Compared with existing state-of-the-art classification and retrieval approaches, the proposed framework demonstrates superior performance while maintaining markedly lower computational complexity. Benchmarking against widely used deep learning architectures—including ResNet50, DenseNet121, VGG16, and Vision Transformer (ViT)—shows that our method achieves higher classification accuracy with substantially fewer parameters (Table 5). Likewise, in the retrieval domain our CBIR system outperforms recently published methods on the same dataset (Table 10), achieving higher mAP and Prec@10 scores.

Beyond raw performance, the unified dual-path design yields practical advantages: shared feature learning reduces redundancy between classification and retrieval, and the combination of adaptive receptive fields and attention enables parameter-efficient learning on a modest dataset. These properties make the approach attractive for deployment on resource-constrained clinical hardware. At the same time, the voting-based CBIR scheme—while improving robustness—incurs additional similarity computations; future work could explore approximate nearest-neighbor search or learned hashing to preserve accuracy while improving scalability.

In terms of computational complexity, the proposed framework achieves a favorable balance between performance and efficiency. Beyond the low parameter count (~ 5.2 million parameters, Table 5), the model leverages the lightweight design of the modified GhostNetV3 backbone. At a standard input resolution of 224 × 224, this architecture requires less than 200 MFLOPs (estimated under standard single-multiply-add FLOP-counting conventions), which is substantially lower than commonly used architectures such as ResNet50 (~ 4 GFLOPs) [43] and DenseNet121 (~ 2.9 GFLOPs) [44]. Although the CBIR component introduces additional similarity computations during inference, these involve lightweight vector distance operations on pre-computed 960-dimensional embeddings and scale efficiently with database size, making the framework suitable for deployment on resource-constrained clinical workstations.

Recent work has begun exploring joint classification–retrieval paradigms for medical image analysis, aiming to bridge diagnostic prediction with case-based interpretability. These include prototype-based retrieval frameworks that incorporate uncertainty modeling and transformer-based dual-path architectures employing cross-attention fusion. While such approaches represent important steps toward integrated diagnostic systems, they often rely on computationally intensive backbones and do not explicitly quantify the consistency between diagnostic predictions and retrieved evidence. In contrast, the proposed framework emphasizes parameter efficiency through a shared backbone and introduces the Classification–Retrieval Agreement Score (CRAS) as a novel quantitative measure of alignment between classification outputs and retrieval results.

Taken together, these results justify the selection of GhostNetV3 as a backbone that balances accuracy, interpretability, and computational efficiency for small-to-moderate clinical imaging datasets.

### Limitations

Despite the strong performance achieved by the proposed dual-path framework, several limitations should be acknowledged. First, the experimental evaluation was conducted on a relatively small publicly available dataset comprising 233 patients. Although strict patient-level stratified cross-validation and rigorous regularization strategies (including dropout, weight decay, and early stopping) were employed to mitigate overfitting and ensure that the reported high accuracy reflects consistent performance across folds rather than memorization, the limited cohort size may restrict generalizability.

Second, the dataset originates from a single public source, and no independent multi-center dataset was available for external classification validation. While the adopted evaluation protocol reduces the risk of data leakage and optimistic bias, the absence of external validation remains a limitation. Future studies should evaluate the framework on multi-center, multi-protocol datasets to further assess robustness across scanners, institutions, and acquisition settings. Third, data augmentation was applied at the slice level to improve model generalization; however, augmentation was performed exclusively on training splits after patient-level separation to prevent information leakage. Despite these precautions, correlations between adjacent MRI slices may still influence model behavior, particularly in small datasets.

With respect to interpretability, the proposed framework primarily provides semantic feature- and case-level explanations through retrieval of similar cases. While CRAS offers a quantitative measure of internal consistency between classification predictions and retrieved reference cases, it does not constitute a direct measure of clinical utility. CRAS should therefore be interpreted as a proxy for algorithmic alignment rather than as a surrogate for radiologist diagnostic confidence or decision accuracy. Formal clinical validation, such as reader studies evaluating diagnostic confidence or interpretation time with and without retrieval support, remains an important direction for future work.

Finally, regarding visual interpretability, this study prioritizes content-based retrieval as a decision-support mechanism rather than pixel-level localization maps. Standard heatmap visualization techniques such as Grad-CAM assume a fixed geometric correspondence between feature maps and input pixels. However, the deformable convolutions employed in the backbone dynamically learn spatial offsets that adjust receptive fields to accommodate irregular tumor morphology. Projecting these shifted features back onto the original image grid using conventional heatmap methods may therefore lead to spatial misalignment. Consequently, this work emphasizes case-based interpretability through retrieval of clinically and histologically similar reference cases, aligning with the comparative diagnostic workflow commonly adopted by radiologists. Future work could explore deformable-aware or feature-aligned visualization techniques or region perturbation analysis to complement retrieval-based interpretability.

## Conclusion

This paper introduced a novel diagnostic framework integrating tumor classification with CBIR to simultaneously enhance diagnostic accuracy and interpretability in brain tumor assessment. Leveraging the strengths of GhostNetV3, a Decoupled Fully Connected (DFC) attention mechanism, and deformable convolutions, our approach minimizes the need for extensive preprocessing while effectively distinguishing between various tumor classes. Extensive experiments on a publicly available T1-weighted contrast-enhanced MRI dataset of 233 patients demonstrated high performance, achieving 99.71% classification accuracy (with precision, recall, and F1-score exceeding 0.99) and strong retrieval effectiveness (mAP: 97.74%, Prec@10: 99.78%), surpassing recently published existing retrieval methods. The robustness of the framework was further evidenced by a novel Classification-Retrieval Agreement Score (CRAS) exceeding 0.96, quantifying the strong synergy between the classification and retrieval components. The integration of CBIR offers clinicians valuable contextual insights, facilitating more informed and confident clinical decision-making.

## Data Availability

The dataset analyzed in this study is publicly available on Figshare ( [https://figshare.com/articles/dataset/brain_tumor_dataset/1512427)) under the Creative Commons Attribution 4.0 International (CC BY 4.0) license. No new datasets were generated. The code and trained models supporting the findings of this study are available from the corresponding author upon reasonable request.

## Abbreviations

CBIR: Content-Based Image Retrieval
CRAS: Classification–Retrieval Agreement Score
DFC: Decoupled Fully Connected
MRI: Magnetic Resonance Imaging
ViT: Vision Transformer
